# A needle-free intradermal PEDV S protein vaccine elicits protective lactogenic immunity in piglets

**DOI:** 10.3389/fcimb.2026.1720440

**Published:** 2026-03-30

**Authors:** Shengjin Liu, Rui Geng, Lu Wang, Xiaona Wei, Yongchang Cao, Hanqin Shen, Feng Chen

**Affiliations:** 1 College of Animal Science, South China Agricultural University, Guangzhou, China; 2 Sate Key Laboratory of Biocontrol, School of Life Sciences, Sun Yat-sen University, Guangzhou, China; 3 School of Life Sciences, Zhengzhou University, Zhengzhou, China; 4 School of Food & Pharmaceutical Engineering, Zhaoqing University, Zhaoqing, Guangdong, China; 5 Guangdong Laboratory for Lingnan Modern Agriculture, Yunfu, China; 6 Guangdong Provincial Enterprise Key Laboratory of Healthy Animal Husbandry and Environment Control, Wen’s Foodstuff Group Co. Ltd, Yunfu, China

**Keywords:** coronavirus vaccines, lactogenic immunity, needle-free intradermal immunization, PEDV, spike protein subunit vaccines

## Abstract

Porcine epidemic diarrhea virus (PEDV) remains a major threat to the global swine industry, with genotype II (GII) strains causing nearly 100% mortality in neonatal piglets. Current vaccination strategies and biosafety control measures provide limited cross-protection and may pose potential biosafety risks, highlighting the urgent need for safer and more effective preventive approaches. In this study, we evaluated a spike (S) protein subunit vaccine administered via needle-free intradermal (ID) immunization and assessed its capacity to induce maternally derived passive protection. ID immunization elicited robust PEDV-specific IgG and neutralizing antibody responses comparable to those induced by conventional intramuscular (IM) injection, with immune responses maintained for more than four months. High levels of IgG and neutralizing antibodies were detected in the serum and colostrum of vaccinated sows, and piglets born to these sows exhibited only mild or no diarrhea following virulent PEDV challenge. Viral shedding and intestinal viral loads were markedly reduced, and intestinal lesions were nearly completely prevented. Overall, this needle-free ID vaccination strategy conferred more than 95% passive protection in neonatal piglets while improving animal welfare and vaccination efficiency. These findings demonstrate that ID delivery of an S protein subunit vaccine represents a promising and practical alternative to conventional IM vaccination for effective PEDV control.

## 1 Introduction

Since 2010, outbreaks of highly pathogenic porcine epidemic diarrhea (PED) caused by porcine epidemic diarrhea virus (PEDV) have occurred worldwide, resulting in substantial economic losses to the global swine industry (Pensaert and de Bouck, 1978; Li et al., 2012). PEDV can infect pigs of all ages; however, neonatal piglets younger than 7 days are particularly susceptible. Infection in these piglets is characterized by severe vomiting, watery diarrhea, dehydration, and mortality rates that can reach up to 100% (Lin et al., 2016; Lou et al., 2024). PEDV is a member of the family Coronaviridae and belongs to the genus Coronavirus. Its genome consists of a single-stranded, positive-sense RNA of approximately 28 kb in length (Diep et al., ; Stevenson et al., 2013). Under electron microscopy, PEDV virions appear spherical, with diameters ranging from approximately 95 to 190 nm, and are surrounded by characteristic spike-like projections on the viral envelope (Song and Park, 2012; Kirchdoerfer et al., 2021).

The spike (S) protein is the most prominent antigen on the surface of PEDV virions and is essential for receptor binding and membrane fusion, making it the primary target of neutralizing antibodies (Wicht et al., 2014; Luo et al., 2024). The PEDV S protein is a large type I transmembrane glycoprotein of approximately 1,386 amino acids that forms homotrimeric spikes on the viral envelope and can be cleaved by host proteases into the S1 and S2 subunits (Tang et al., 2020; Qin et al., 2024). Phylogenetic analyses based predominantly on sequence variation within the S1 subunit classify PEDV strains into two major genotypes, G1 and G2, which differ substantially in amino acid composition within this region (Jung et al., 2020; Zhang et al., 2023). Notably, multiple key neutralizing epitopes are present in both the S1 and S2 subunits, including epitopes in the S1 region such as S1A and COE, as well as epitopes in the S2 region such as SS2, SS6, and 2C10 (Sun et al., 2008; Okda et al., 2017; Kong et al., 2020). The degree of conservation of these epitopes varies between genotypes, which may limit cross-neutralization (Chen et al., 2016; Zhuang et al., 2022). However, several critical epitopes within the S1 and S2 regions remain conserved across different strains, supporting the use of the full S ectodomain as a vaccine antigen (Fu et al., 2017).

Moreover, previous studies have demonstrated the feasibility of S protein-based subunit vaccines, including our own evaluation of passive immunity in piglets born to vaccinated sows (Liu et al., 2025). Alternative S-targeting strategies, such as AAV-mediated S protein expression and cell membrane nanovesicle-based S vaccines, have also shown favorable immunogenicity and protective efficacy, further supporting the rationale for S protein-based vaccine development (Pang et al., 2025; Wang et al., 2026). Therefore, in this study, we selected the PEDV S ectodomain protein previously designed and expressed in our laboratory as the antigen for subunit vaccine preparation and immunogenicity evaluation.

With respect to immunization routes, IM injection remains the most commonly used method in commercial swine production (Turner et al., 2011; Opriessnig et al., 2021). Nevertheless, this approach is associated with several disadvantages, including increased stress responses, labor-intensive handling, and the risk of disease transmission through needle reuse (Ferre et al., 2005; Dalmau et al., 2021; Madapong et al., 2021). In recent years, needle-free ID immunization has gained increasing attention because of its high efficiency, improved biosafety, and ease of administration (Laurent et al., 2007; Romani et al., 2010; Hickling et al., 2011). Previous studies have demonstrated that needle-free delivery of several commercial swine vaccines can induce antibody responses comparable to those achieved by IM injection (Houser et al., 2004; Temple et al., 2017; Temple et al., 2020). Therefore, the development of vaccines suitable for ID immunization represents an important strategy for improving vaccination efficiency and biosafety in modern swine production systems.

In this study, we systematically evaluated the immunogenicity of a PEDV S protein subunit vaccine delivered via a needle-free injection system. Antibody-mediated and cell-mediated immune responses were assessed to compare immune activation elicited by needle-free ID and conventional IM administration of the vaccine. In addition, neonatal piglets born to immunized sows were challenged with PEDV, and clinical signs, viral shedding, and intestinal pathological changes were evaluated to determine whether needle-free ID vaccination could serve as an effective alternative to traditional IM vaccination for PEDV prevention.

## 2 Materials and methods

### 2.1 Cells and virus

Vero cells were maintained in Dulbecco’s Modified Eagle Medium (DMEM) supplemented with 10% fetal bovine serum (FBS; Gibco, USA) at 37 °C in a humidified incubator with 5% CO_2_. The PEDV strain hddz (GenBank accession no. PQ316088) was propagated in Vero cells in DMEM containing 7 µg/mL trypsin, and viral stocks were stored at −80 °C for subsequent experiments.

### 2.2 Preparation of PEDV subunit vaccine

The spike protein of PEDV (strain dndz) was obtained as previously described (Liu et al., 2025). The gene encoding the S-ECD (amino acids 1–1325), fused with a C-terminal T4 foldon, was cloned into the pMGT vector (Cantonbio, China) containing a glutamine synthetase (GS) selection marker. The plasmid was transfected into CHOZN^®^ CHOK1 cells using polyethyleneimine (PEI) at a mass ratio of 1:1.5. Stable cell pools were generated by selection in EXCELL^®^ CD CHO Fusion Medium (Sigma) supplemented with 25 μM methionine sulfoximine (MSX). Once cell viability exceeded 90%, MSX was removed and the cell pool was inoculated into EXCELL^®^ Advanced CHO Fed-batch Medium (Sigma) at a density of 0.5 ×10^6^ cells/mL. Starting from day 3, the culture was fed daily with 3% (v/v) HyClone^®^ Cell Boost 7a and 0.3% (v/v) Cell Boost 7b. The culture supernatant was harvested when cell viability dropped below 90% and clarified by centrifugation at 4000 ×g for 20 min.

The spike protein was captured from the culture supernatant using a Q Focurose FF column (Huiyan-bio) pre-equilibrated with 20 mM phosphate buffer (PB, pH 7.0) and eluted with a linear gradient of 0–0.5 M NaCl. The eluted fractions were analyzed by SDS-PAGE, and those containing the target protein were pooled and concentrated by ultrafiltration prior to further purification by size-exclusion chromatography (SEC). The concentrated sample was then loaded onto a Superdex 200 (16/60) column (GE Healthcare) pre-equilibrated with PBS (pH 7.4), and fractions were collected at a flow rate of 1 mL per tube. Fractions with a purity greater than 90% were pooled for subsequent animal experiment.

The purified S protein was emulsified with MONTANIDE™ ISA 201 VG adjuvant (SEPPIC, France) at a 1:1 (w/w) ratio to prepare the subunit vaccine. Briefly, the S protein was diluted in PBS to the target concentration, and both the protein solution and adjuvant were preheated to 35 °C. Under continuous magnetic stirring, the aqueous phase was slowly added to the oil phase to form a stable emulsion. The mixture was then stirred at 35 °C, allowed to stand at 4 °C, and returned to room temperature before being used for immunization.

### 2.3 Immunization of piglets

Thirty clinically healthy, weaned Duroc× Landrace × Yorkshire (DLY) crossbred piglets (28 days of age, mixed sex) were sourced from a PEDV-free commercial herd and confirmed to be seronegative for PEDV-specific antibodies by ELISA before the study. The animals were randomly allocated into five groups, including one PBS control group, two IM groups, and two ID groups receiving either 50 µg/dose or 75 µg/dose of the vaccine.

ID immunization was performed using a commercial needle-free injector (NDI-BP-ED1, Lwscenter, China) delivering 0.2 mL per dose. piglets in the PBS control group received sterile PBS via the same ID route. IM injections were administered using conventional syringes. Both ID and IM immunizations were delivered in the neck region posterior to the ear base.

Each group was administered a primary immunization on day 0 followed by a booster on day 14. Serum samples were collected weekly to measure S protein–specific IgG and neutralizing antibody titers throughout the fattening period until the animals reached the standard age for marketing.

### 2.4 Protective efficacy of passive immunity against challenge infection

Twelve PEDV-negative pregnant primiparous sows (commercial Duroc × Landrace crossbred) were randomly assigned into three groups, including PBS, an IM group receiving 75 µg/dose, and an ID group receiving 75 µg/dose. Immunizations were administered at 28 days (prime) and 14 days (booster) before parturition. Serum and colostrum samples were collected at designated time points for the analysis of IgG levels and neutralizing antibody responses. At 5 days post-parturition, all piglets were orally challenged with the PEDV hddz strain (1×10^6^ tissue culture infectious dose [TCID_50_] per dose). Clinical signs (diarrhea, vomiting, anorexia, and depression) were assessed daily for 7 consecutive days. Diarrhea scores and viral shedding were monitored daily based on anal swab samples collected for viral shedding detection. Diarrhea severity was scored from 0 to 4 according to fecal consistency and clinical status: solid (0), pasty (1), semi-liquid (2), liquid (3), and liquid feces with depression (4). On 3 days post-challenge (3 DPC), three piglets from each group were humanely euthanized for necropsy and tissue collection. Deep anesthesia was induced by intravenous administration of sodium pentobarbital (100 mg/kg body weight) until loss of consciousness and absence of pedal withdrawal reflex were confirmed, after which euthanasia was completed by pentobarbital overdose in accordance with institutional animal care and use guidelines. Intestinal tissues, including duodenum, jejunum, and ileum, were aseptically collected for viral load quantification and histopathological examination.

### 2.5 Indirect ELISA

PEDV S protein-specific IgG was measured using commercial ELISA kits (DHN, China) according to the manufacturer’s protocols. The microplates provided in the kit were pre-coated with recombinant PEDV S protein derived from a genotype G2c strain. Briefly, diluted serum samples (100 µL/well) were incubated at 37 °C for 1 h, washed three times, and incubated with HRP-conjugated secondary antibody for 1 h. After washing, substrate solution was added and incubated at 37 °C for 10 min in the dark. The reaction was terminated, and absorbance was measured at 450 nm. The results were expressed as the S/P (sample-to-positive) ratio, which represents the relative antibody level of each sample compared with the positive control provided in the kit, and was calculated using the following formula: S/P = (OD_experimental_ - OD_negative_)/(OD_positive_ - OD_negative_).

### 2.6 Neutralizing antibody assay

Neutralizing antibody titers were determined following the method described by Hu et al (Hu et al., 2025). Serum and colostrum samples were heat-inactivated at 56 °C for 30 min, twofold serially diluted, and incubated with an equal volume of PEDV inoculum (200 TCID_50_). After incubation at 37 °C for 1 h, the mixtures were inoculated onto Vero cells for 2 h. The inoculum was then replaced with maintenance medium containing 7 µg/mL trypsin and incubated for 72 h. Cytopathic effect (CPE) was observed, and the neutralizing antibody titer was defined as the highest dilution capable of inhibiting 50% of CPE.

### 2.7 Lymphocyte proliferation assay

At day 14 post-boost, peripheral blood mononuclear cells (PBMCs) were isolated using a porcine peripheral blood mononuclear cell isolation kit (Solarbio, China) according to the manufacturer’s instructions. Then, PBMCs were seeded into 96-well plates at 1×10^5^ cells per well in RPMI-1640 medium supplemented with 10% fetal bovine serum (FBS) and penicillin–streptomycin. To specifically evaluate vaccine antigen–matched cellular recall responses, PBMCs were stimulated with recombinant PEDV S protein. Cells were stimulated with 10 µg/mL S protein (experimental group), RPMI-1640 medium (negative control), or 10 µg/mL ConA (positive control), respectively. After incubation at 37 °C with 5% CO_2_ for 72 h, 10 μL of Cell Counting Kit-8 (CCK-8) solution (NCM Biotech, China) was added to each well and incubated for an additional 2 h. Wells containing only cells and medium served as blanks. Each sample was tested in triplicate. Absorbance was measured at 450 nm using a microplate reader. Lymphocyte proliferation was indirectly evaluated based on metabolic activity, and results were expressed as stimulation index (SI). The stimulation index (SI) was calculated as: SI = (OD_experimental_ - OD_blank_)/(OD_negative_ - OD_blank_).

### 2.8 Detection of IFN-γ and IL-4

PBMCs were seeded at 1×10^5^ cells per well in the same RPMI-1640 culture medium and stimulated with 10 µg/mL S protein for 72 h, with unstimulated cells cultured in parallel as negative controls. Supernatants were harvested by centrifugation, and cytokine concentrations (IFN-γ and IL-4) were measured using commercial ELISA kits (Meimian, China) according to the manufacturer’s instructions. Values were calculated based on the corresponding standard curves.

For the IFN-γ ELISA, the limit of detection (LOD) was 0.1 pg/mL and the limit of quantification (LOQ) was 1.25 pg/mL. For the IL-4 ELISA, the LOD was 0.1 pg/mL and the LOQ was 2 pg/mL. Values below the LOD were assigned the LOD value for statistical analysis.

### 2.9 Real-time quantitative PCR

PEDV RNA was detected by real-time quantitative PCR (RT-qPCR) in accordance with the MIQE guidelines. Total RNA was extracted from piglet rectal swabs and homogenized intestinal tissues using the RNeasy Mini Kit (Qiagen, Germany). RNA concentration and purity were assessed using a NanoPhotometer spectrophotometer (Germany), and only samples with A260/A280 ratios between 1.8 and 2.1 were used for further analysis. After normalization of nucleic acid concentrations, a fixed amount of total RNA (500 ng) was subjected to reverse transcription using the SuperScript III First-Strand Synthesis Kit (Vazyme, China).

Quantitative PCR was performed using a TaqMan-based RT-qPCR assay targeting the PEDV N gene in a total reaction volume of 20 μL. The primer sequences were as follows: forward primer, 5′-GAATTCCCAAGGGCGAAAAT-3′; reverse primer, 5′-TTTTCGACAAATTCCGCATCT-3′. The probe sequence was 5′-FAM-CGTAGCAGGCTTGCTTCGGACCCA-BHQ-3′. Primers and probe were synthesized by Sangon Biotech (Shanghai, China) and supplied as stock solutions at a concentration of 100 μM. Amplification was carried out using a commercial TaqMan qPCR Master Mix containing Taq DNA polymerase (Yeasen, China), added according to the manufacturer’s instructions.

Thermal cycling conditions consisted of an initial denaturation at 95 °C for 20 s, followed by 40 cycles of denaturation at 95 °C for 3 s and annealing/extension at 60 °C for 30 s. Reactions were performed on a 7500 Real-Time PCR System (Thermo Fisher Scientific, USA). No-template controls were included in each run, and quantification was performed based on a previously established standard curve.

### 2.10 Histopathology and immunohistochemistry

Intestinal tissues were fixed in 4% formaldehyde for 48 h at room temperature, embedded in paraffin, and sectioned at a thickness of 3–4 μm. Sections were subjected to hematoxylin and eosin (H&E) staining for histopathological evaluation. For immunohistochemistry (IHC) analysis, paraffin sections were deparaffinized and rehydrated. Endogenous peroxidase activity was blocked using 3% hydrogen peroxide, followed by washing with PBS. Sections were incubated with a mouse monoclonal antibody against the PEDV nucleocapsid (N) protein (Zoonogen, China) at a dilution of 1:200. Subsequently, sections were incubated with an HRP-conjugated goat anti-mouse IgG secondary antibody (Beyotime, China) at a dilution of 1:200. Immunoreactive signals were visualized according to the manufacturer’s instructions.

### 2.11 Statistical analysis

All statistical analyses were performed using GraphPad Prism 10 (GraphPad Software, USA). Data are presented as mean ± standard deviation (SD). Prior to one-way analysis of variance (ANOVA), data were assessed for normality using the Shapiro–Wilk test and for homogeneity of variances using Levene’s test. One-way ANOVA was applied when these assumptions were met. Statistical significance was defined as *P < 0.05, **P < 0.01, and ***P < 0.001.

## 3 Results

### 3.1 Needle-free ID immunization with the PEDV spike protein subunit vaccine induces strong humoral responses

**Figure 1:**
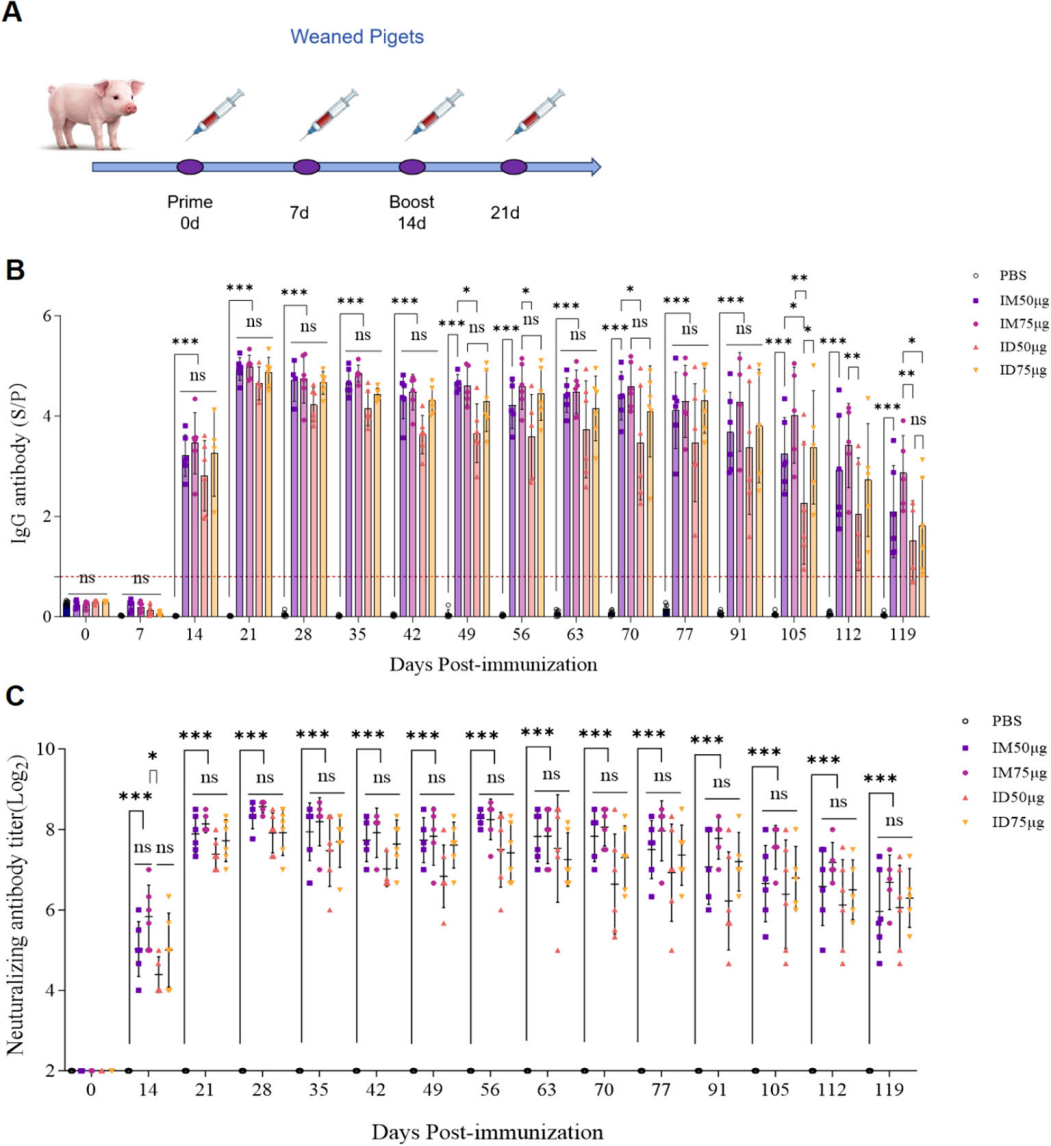
Humoral immune responses in piglets immunized with the PEDV-S subunit vaccine via two delivery routes. **(A)** Experimental design and sampling schedule. Syringe icons indicate the time points of sample collection. **(B)** Serum PEDV-specific IgG levels measured by ELISA (sera diluted 1:300). The dotted line indicates the positivity cutoff (S/P = 0.8). **(C)** Serum neutralizing antibody (NAb) titers measured by virus neutralization assay in Vero cells. Twofold serially diluted sera were incubated with PEDV (200 TCID_50_), and titers were defined as the highest dilution preventing cytopathic effects. Data are presented as mean ± SD (n = 6 per group). Statistical significance: ns, *P < 0.05, **P < 0.01, and ***P < 0.001.

To evaluate the immunogenicity of the PEDV S protein subunit vaccine delivered via needle-free ID injection, serum samples from immunized piglets were analyzed by ELISA for PEDV S-specific IgG antibody, and neutralizing antibody titers against PEDV were determined (**Figure 1A**). Following primary immunization, PEDV S-specific IgG levels increased markedly in vaccinated piglets. One week after the booster, PEDV S-specific IgG level reached their peak and were significantly higher than those in the control group. Notably, the high antibody levels induced by both immunization routes were sustained for up to four months post-immunization (**Figure 1B**).

Neutralizing antibodies are critical for protection against PEDV infection. The neutralizing antibody titers in the ID group were comparable to those in the IM group, with mean titers exceeding 1:256 and significantly higher than those in controls (**Figure 1C**). No significant differences were observed between the ID and IM groups, indicating that both routes effectively induced robust humoral immune responses.

**Figure 2:**
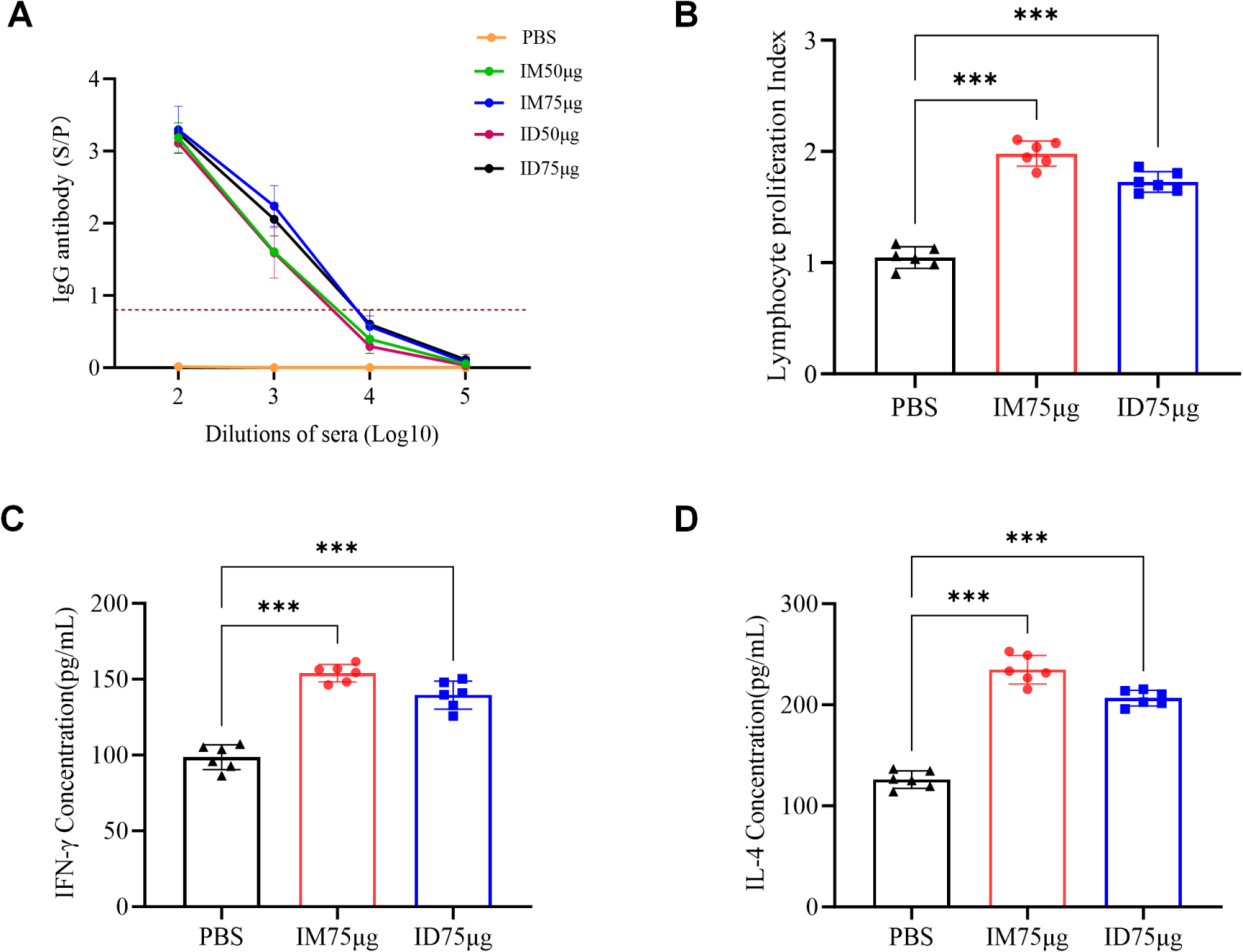
Vaccine-induced humoral and cellular immune responses in piglets. **(A)** Serum PEDV-specific IgG antibody titers determined by 10-fold serial dilution ELISA. The dotted line represents a serum ratio of 0.8, above which samples were considered positive. **(B)** PBMC proliferation-associated metabolic activity measured by CCK-8 assay. PBMCs were isolated at day 14 post-boost, cultured in RPMI-1640 medium, and stimulated with PEDV-S protein (10 µg/mL) for 72 h. **(C)** IFN-γ levels in PBMC culture supernatants quantified by ELISA following PEDV-S protein stimulation. **(D)** IL-4 levels in PBMC culture supernatants quantified by ELISA following PEDV-S protein stimulation Data are shown as mean ± SD (n = 6 per group). Statistical significance: ***p < 0.001.

Gradient dilution assays performed on sera collected two weeks after the booster revealed that in the 50 μg group, IgG antibodies became undetectable at a dilution of 10^4^, whereas the ID 75 μg group remained positive at this dilution, with rates comparable to those in the IM 75 μg group (**Figure 2A**). Together, these findings indicate that needle-free ID delivery of the PEDV-S subunit vaccine induces strong and durable IgG and neutralizing antibody responses. Moreover, its immunogenicity is equivalent to that of conventional IM injection, the 75 μg dose tended to induce higher IgG and neutralizing antibody levels than the 50 μg dose and was therefore used in subsequent experiments.

### 3.2 Analysis of lymphocyte proliferation and cytokine secretion

Lymphocyte proliferation was assessed two weeks after the booster immunization. PBMCs isolated from immunized piglets were cultured in RPMI-1640 medium and stimulated with the PEDV S protein (10 μg/mL) for 72 h. Both ID and IM administration of the PEDV S protein subunit vaccine enhanced antigen-specific lymphocyte proliferative responses compared with the control group (**Figure 2B**). To further characterize vaccine-induced cellular responses, cytokine levels were measured by ELISA. All immunized piglets exhibited similarly elevated concentrations of IFN-γ (approximately 150 pg/ml) and IL-4 (approximately 200 pg/ml) (**Figures 2C, D**). These data demonstrate that both ID and IM immunization effectively promoted lymphocyte activation and cytokine secretion.

### 3.3 Humoral immune responses in sows following needle-free ID immunization

**Figure 3:**
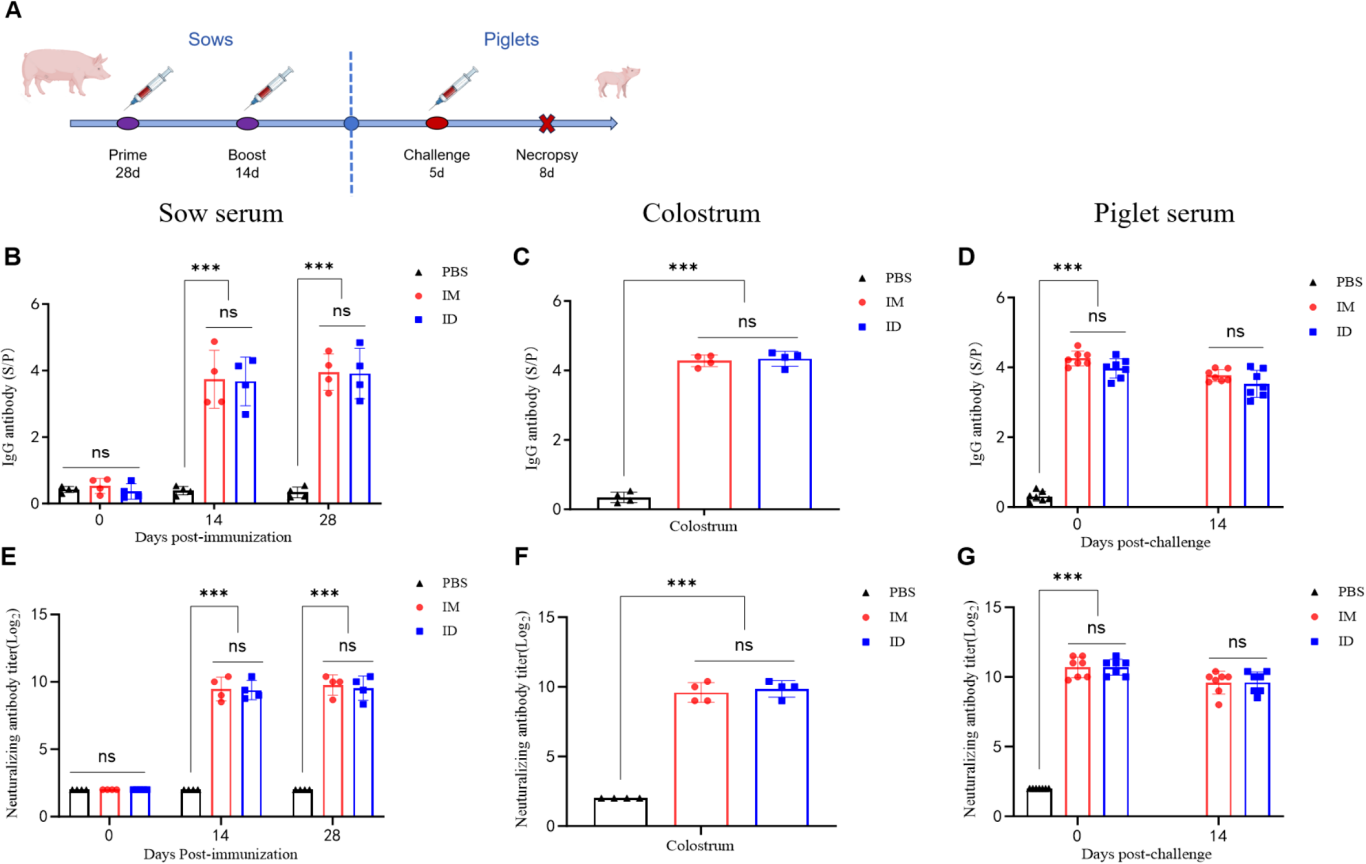
Humoral immune responses in sows immunized with the PEDV-S subunit vaccine and passive antibody transfer to their piglets. **(A)** Schematic representation of the experimental design. Syringe icons indicate the time points of sample collection. **(B–D)** PEDV S-specific IgG antibody levels in sow serum, piglet serum, and colostrum determined by ELISA. **(E–G)** Neutralizing antibody titers in sow serum, piglet serum, and colostrum determined by virus neutralization assay using Vero cells. Twofold serially diluted sera were incubated with PEDV (200 TCID_50_), and titers were defined as the highest dilution preventing cytopathic effects. Data are presented as mean ± SD (n = 4 sows or n = 7 piglets per group). Statistical significance: ns, ***p < 0.001.

The immunogenicity of the PEDV Spike protein subunit vaccine delivered by needle-free ID injection in sows was compared with conventional IM administration (**Figure 3A**). Serum samples were analyzed by ELISA for PEDV-specific IgG. All animals were confirmed seronegative prior to vaccination and were randomly allocated to ensure comparable baseline conditions across groups. Both ID and IM immunization induced robust IgG responses in serum and colostrum (**Figures 3B, C**), with no significant differences between the two routes, indicating that needle-free ID vaccination provides immunogenicity in sows equivalent to IM injection.

Further analysis revealed that both delivery routes elicited high titers of neutralizing antibodies in sow serum and colostrum (approximately 1:1024) (**Figures 3E, F**). Prior to viral challenge, high levels of IgG were already detectable in piglet serum at three days of age, indicating efficient transfer of maternal antibodies from sows to piglets via colostrum (**Figure 3D**). In addition, neutralizing antibodies were detected in piglet serum, with titers exceeding 1:512 (**Figure 3G**). Importantly, antibody levels were still maintained at high levels two weeks later in both groups. Collectively, these results demonstrate that needle-free ID immunization in sows effectively stimulates high levels of specific antibodies and ensures their passive transfer to piglets, thereby supporting enhanced maternal immunity and neonatal protection.

### 3.5 Protective efficacy of maternal immunity in piglets

**Figure 4:**
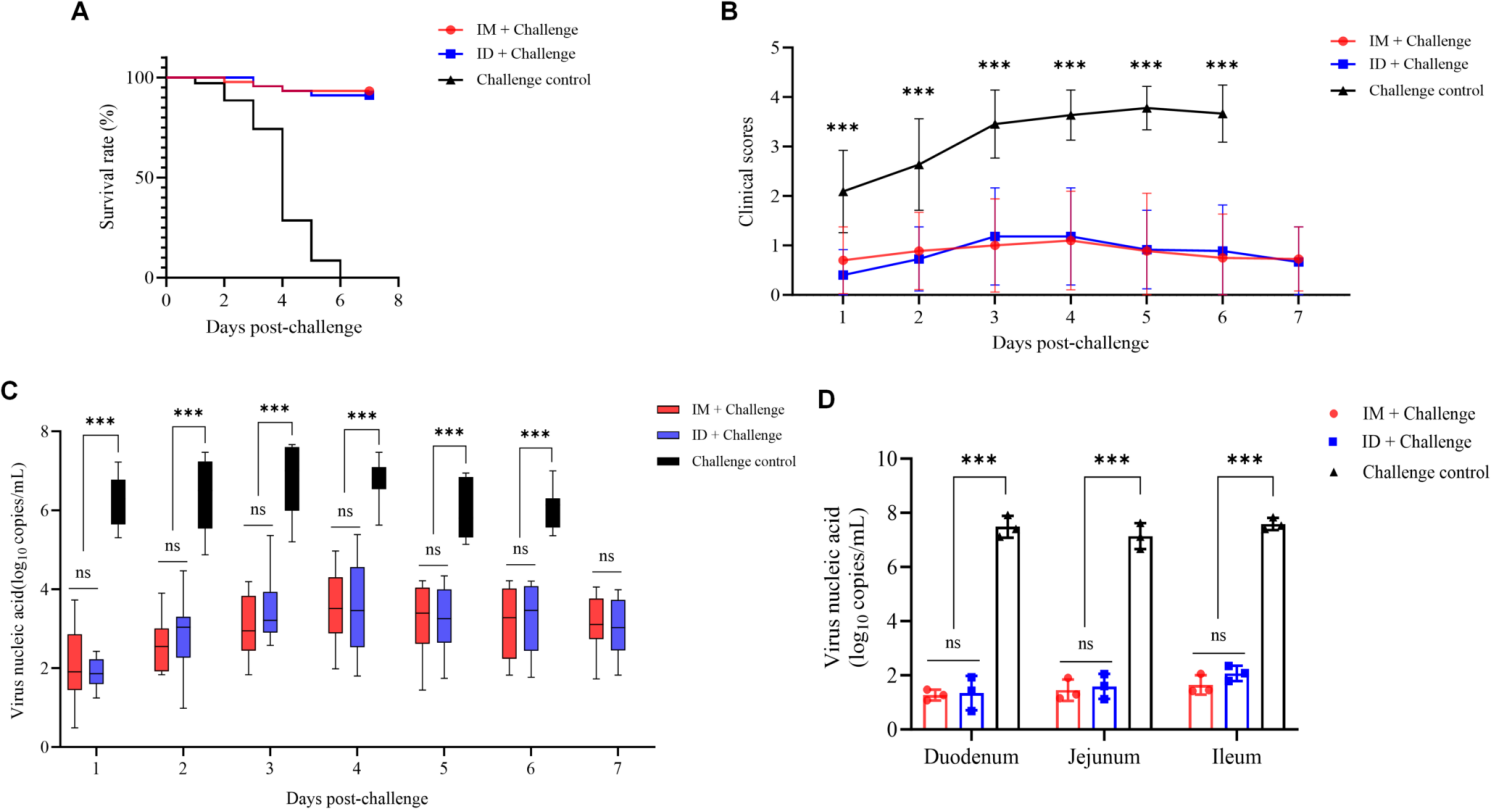
Protective efficacy in neonatal piglets. Piglets from the ID, IM and control groups were all challenged with PEDV. **(A)** Survival rates following viral challenge. **(B)** Diarrhea scores recorded daily from 1 to 7 DPC. **(C)** Viral shedding in anal swab samples quantified by RT-qPCR. **(D)** Viral RNA copies in intestinal tissues collected at 3 DPC determined by RT-qPCR. RNA was extracted from anal swab samples and homogenized intestinal tissues. Data are presented as mean ± SD (n = 10 piglets per group). Statistical significance: ns, ***p < 0.001.

To assess the protective efficacy of ID immunization and IM immunization, 5-day-old piglets were challenged with the PEDV hddz strain, a highly virulent and currently prevalent G2c genotype PEDV strain. In the challenge control group, piglets developed vomiting and watery-to-mild diarrhea as early as 1 day post-challenge (DPC). Mortality was observed at 3 DPC, with nearly all piglets exhibiting severe diarrhea, malnutrition, and poor body condition; all animals succumbed by 6 DPC (**Figure 4A**). In contrast, vaccinated piglets remained clinically healthy throughout the study, exhibiting no signs of vomiting, dehydration, lethargy, or anorexia, with only a few less robust individuals developing mild diarrhea (**Figure 4B**).

To further assess viral replication and shedding, PEDV genomic copy numbers were quantified from fecal swabs collected daily from 1 to 7 DPC. In the challenge control group, viral loads in fecal swab suspensions rapidly reached 10^6^–10^8^ copies/mL, peaking at 3 DPC. By contrast, both the needle-free ID and IM groups maintained viral loads below 10^4^ copies/mL, with peak levels observed at 4–5 DPC. Across the observation period, virus shedding was significantly suppressed in both vaccinated groups compared with controls (P < 0.001) (**Figure 4C**).

To examine tissue viral distribution, three piglets per group were euthanized at 3 DPC, and intestinal tissues were collected. PEDV RNA loads were measured in the duodenum, jejunum, and ileum of challenged piglets. Viral RNA was detectable in all sampled intestinal segments; however, vaccinated groups exhibited markedly reduced levels compared with challenge control (**Figure 4D**). No significant differences were observed between the ID and IM groups, both of which demonstrated strong inhibitory effects on viral replication.

### 3.6 Gross pathology, histopathology, and immunohistochemical analysis

**Figure 5:**
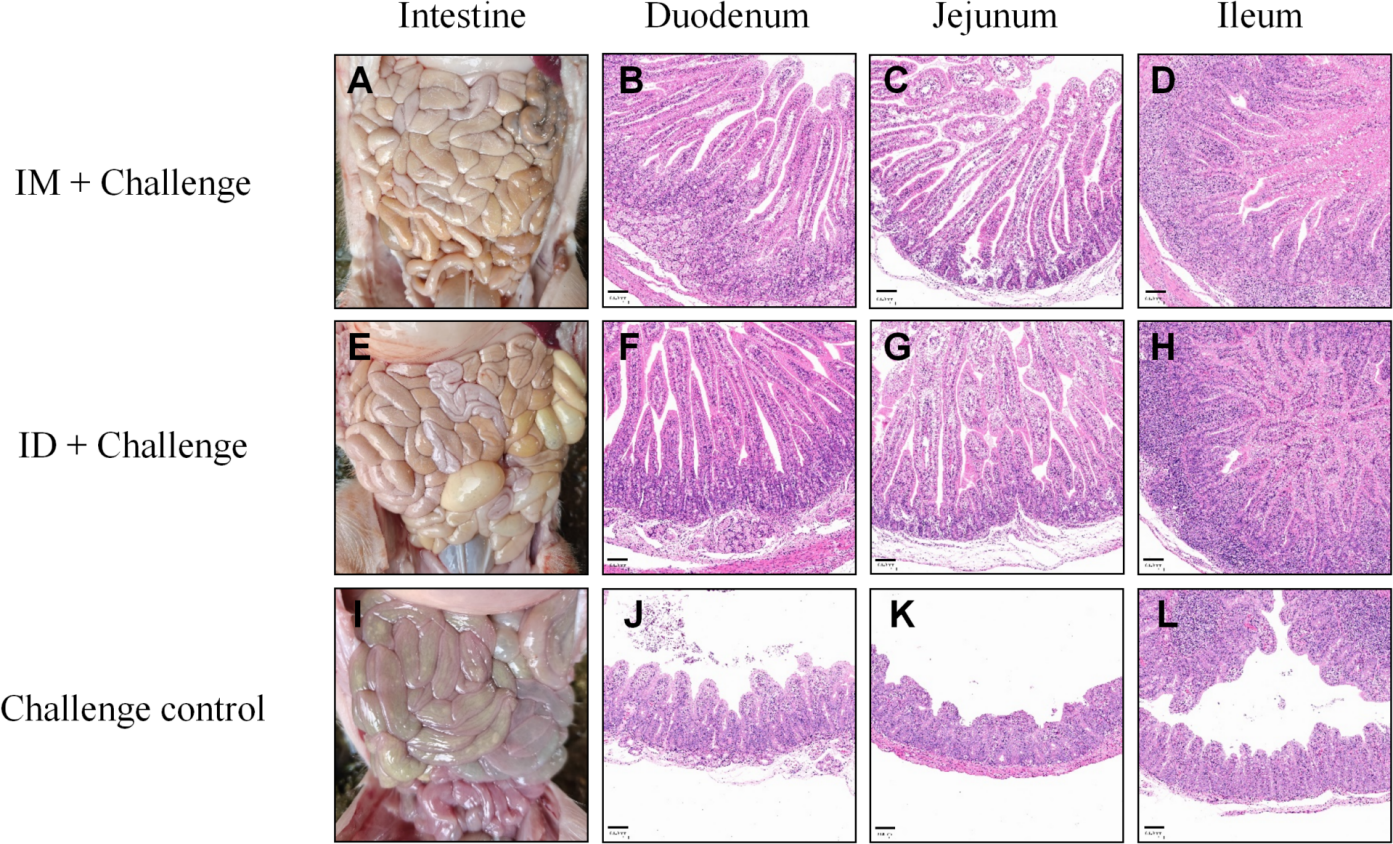
Gross and histopathological examination of piglet intestines at 3 DPC following PEDV challenge. Representative intestinal tissues were collected at necropsy and stained with H&E. **(A–D)** represent the IM + challenge group, **(E–H)** represent the ID + challenge group, and **(I–L)** represent the challenge control group. Scale bar = 100 μm.

To further evaluate vaccine-mediated protection, the same three piglets per group euthanized at 3 DPC were subjected to necropsy and histological analysis. Gross pathological examination revealed intact intestinal morphology in piglets born from vaccinated sows, characterized by normal structure and bright red coloration without visible lesions (**Figures 5A, E**). These findings indicated that vaccination effectively prevented PEDV-induced intestinal damage. In contrast, challenged control piglets exhibited classical PEDV-associated lesions, including thinning of the intestinal wall, increased permeability, gaseous distension, and accumulation of yellow watery contents, consistent with severe enteritis and fluid retention (**Figure 5I**).

Histopathological examination further corroborated these observations. Challenged control piglets displayed severe jejunal and ileal lesions, including villous atrophy and blunting, vacuolar degeneration of epithelial cells, crypt destruction, focal necrosis, and epithelial shedding. Additional features included submucosal edema, central lacteal dilation, and crypt dysplasia; in some regions, villi were completely lost, leaving only thin remnants of the intestinal wall (**Figures 5J, K, L**). In contrast, piglets born from vaccinated sows preserved normal intestinal architecture, with intact villi, maintained villus height-to-crypt depth ratios, and no epithelial shedding or pronounced inflammatory infiltration (**Figures 5B–D, F–H**). These findings demonstrated that both ID and IM immunization preserved intestinal integrity and prevented PEDV-induced histological damage.

**Figure 6:**
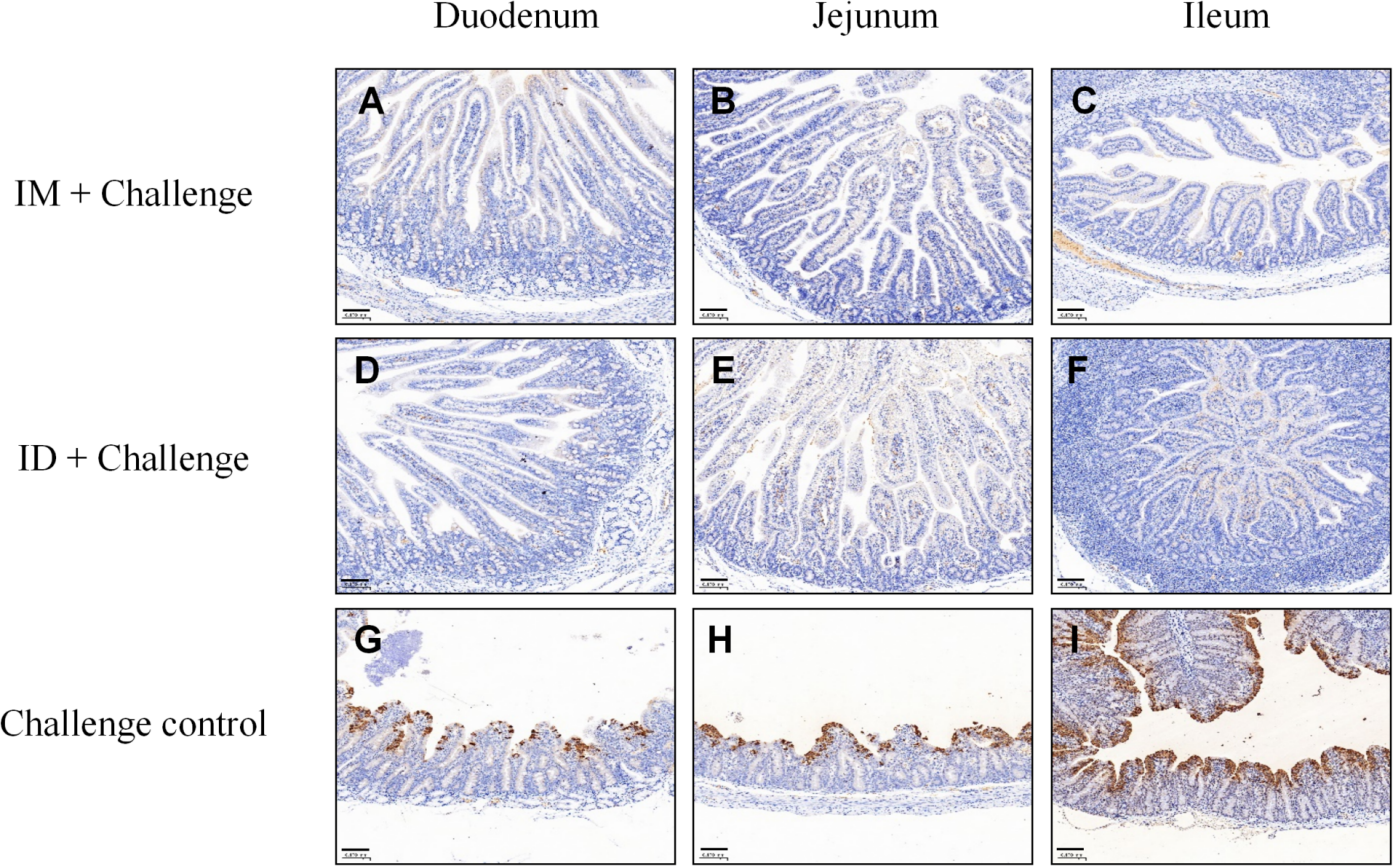
Immunohistochemical (IHC) detection of PEDV antigen in piglet intestines at 3 DPC. Representative intestinal sections were subjected to IHC staining for the PEDV N antigen. **(A–C)** represent the IM + challenge group, **(D–F)** represent the ID + challenge group, and panels **(G–I)** represent the challenge control group. Scale bar = 100 μm.

Immunohistochemistry (IHC) further supported these results. In piglets born from unvaccinated sows, diffuse granular PEDV N protein antigen signals were detected in the cytoplasm of jejunal and ileal enterocytes, predominantly at villus tips, colocalizing with regions of villous atrophy and structural destruction (**Figures 6G–I**). Scattered antigen-positive signals were also observed in submucosal lymphoid tissues, indicating extensive viral replication and dissemination. In contrast, no PEDV antigen was detected in vaccinated piglets, whose intestinal mucosa remained intact and showed no pathological staining (**Figures 6A–F**).

Collectively, gross pathology, histopathology, and IHC consistently demonstrated that the needle-free ID PEDV S protein subunit vaccine conferred protective efficacy equivalent to IM immunization in piglets born from vaccinated sows. Both routes effectively preserved intestinal structure and function while restricting viral replication and dissemination in these piglets.

## 4 Discussion

Since the emergence of highly virulent PEDV variants, the virus has remained a major challenge to global swine production, particularly due to its high morbidity and mortality in neonatal piglets (Jung and Saif, 2015; Zhang et al., 2023). Although inactivated and live-attenuated vaccines have been widely applied, disease outbreaks continue to occur, underscoring the need for safer and more effective vaccination strategies (Langel et al., 2016; Wang et al., 2024). The ID route enables precise antigen delivery to the dermis, a tissue enriched in antigen-presenting cells such as Langerhans cells and dendritic cells (Nfon et al., 2008; Nicolas and Guy, 2008). In addition, this delivery route was associated with practical advantages, including reduced injection-associated stress, elimination of needle-related risks, and improved handling efficiency (Zempsky et al., 2016). Collectively, these observations suggest that ID immunization represents a feasible alternative vaccination approach that balances immunogenicity, biosafety, and animal welfare in swine production.

Previous studies on PEDV S protein subunit vaccines have primarily focused on comparisons with inactivated vaccines, optimization of antigen delivery platforms and adjuvant systems, or enhancement of immune durability (Liu et al., 2025; Pang et al., 2025; Wang et al., 2026). Despite differences in experimental design, these studies consistently support the feasibility of the S protein subunit as a PEDV vaccine antigen and demonstrate its capacity to induce immune responses in sows, promote antibody production, and confer passive protection to piglets. In the present study, we evaluated the impact of immunization route on vaccine-induced immunity by comparing needle-free ID administration with conventional IM injection. This comparison provides additional insight into the influence of vaccine delivery strategies on antibody levels, the efficiency of maternal antibody transfer, and associated protective outcomes. Collectively, the results indicate that, when the same vaccine antigen is used, needle-free ID immunization can induce protective efficacy comparable to that achieved by IM injection.

Immunogenicity analyses revealed that the needle-free ID route induced seroconversion within 14 days post-immunization, with antibody titers significantly elevated by day 21, accompanied by robust neutralizing activity (**Figure 1**). Further assessment showed that both IM and ID routes at an antigen dose of 75 μg induced high-level antibody responses persisting for more than four months. The ID immunization group exhibited pronounced lymphocyte proliferation accompanied by elevated secretion of IFN-γ and IL-4 (**Figure 2**). Collectively, these findings demonstrate that the ID delivery route achieves immunogenicity comparable to IM administration.

To further evaluate protective efficacy, we employed a piglet challenge model. Previous studies have established that serum neutralizing antibodies are critical mediators of maternal passive protection (Won et al., 2020; Shi et al., 2021). Our results showed that ID-immunized sows exhibited significantly higher levels of IgG and neutralizing antibodies compared to controls, with neutralizing antibody titers exceeding 1:512 (**Figure 3**, P < 0.001). Piglets from ID-vaccinated sows displayed no signs of diarrhea (clinical score <1), whereas challenged control piglets showed a 100% diarrhea incidence (score ≥3). Viral load analysis revealed nearly a 4-log reduction in PEDV copies in feces from the ID group (**Figure 4**). Histopathological evaluation demonstrated preserved villus height/crypt depth ratios and absence of intestinal lesions in immunized piglets, in contrast to severe villous atrophy in challenged controls (**Figure 5**). Moreover, IHC and RT-qPCR confirmed the absence of PEDV antigen in the intestines of vaccinated piglets, with viral RNA loads significantly reduced compared to challenge controls, indicating a passive protection rate exceeding 95%. Collectively, these results demonstrate that needle-free ID vaccination establishes a strong mucosal-systemic immune barrier, providing effective maternal-derived protection for neonatal piglets.

Together, these findings provide strong experimental support for the needle-free ID vaccination strategy. In addition to demonstrating comparable performance to IM injection, this approach offers multiple added benefits in terms of animal welfare, production efficiency, and biosafety. It should be noted that the protective efficacy of the vaccine was evaluated only in 5-day-old piglets, and challenge experiments were not conducted at later age stages; therefore, the duration of protective immunity cannot be conclusively determined. Moreover, the *in vivo* protective efficacy against different PEDV genotypes was not systematically assessed, and the cross-genotypic protective potential of this vaccine against genetically distinct PEDV strains remains to be further validated. Future studies should focus on large-scale field validation, systematic evaluation of the duration of protection, and assessment of cross-protective efficacy against diverse PEDV genotypes. Such efforts will further substantiate the practical feasibility of this immunization strategy and facilitate the transition toward needle-free and precision-based vaccination approaches in modern swine health management.

## 5 Conclusion

In conclusion, this study demonstrates that a PEDV S protein subunit vaccine delivered via needle-free intradermal immunization is a promising and practical approach for PEDV prevention and control. This vaccination strategy induced robust and durable humoral immune responses and significantly enhanced maternal antibody transfer, conferring >95% passive protection in neonatal piglets. Overall, needle-free intradermal immunization represents a viable alternative to conventional intramuscular vaccination for PEDV control in swine production.

## Data Availability

The original contributions presented in the study are included in the article/supplementary material. Further inquiries can be directed to the corresponding authors.
